# Understanding the Relationships between Landscape Eco-Security and Multifunctionality in Cropland: Implications for Supporting Cropland Management Decisions

**DOI:** 10.3390/ijerph20031938

**Published:** 2023-01-20

**Authors:** Fang Tang, Yangbing Li, Xiuming Liu, Juan Huang, Yiyi Zhang, Qian Xu

**Affiliations:** 1 School of Geography and Environmental Sciences, Guizhou Normal University, Guiyang 550001, China; 2 State Key Laboratory of Environmental Geochemistry, Institute of Geochemistry, Chinese Academy of Sciences, Guiyang 550081, China

**Keywords:** cropland, landscape ecological security, multifunctionality, coupling coordination degree, karst trough valley area

## Abstract

Cropland is an essential strategic resource, for which landscape ecological security and multifunctionality evolution are related to regional stability and sustainable social development. However, few studies have explored the spatial heterogeneity of the coupling between the two from a multiregional and systematic perspective, and the interaction mechanisms have still not been thoroughly analyzed. In this study, a typical karst trough and valley area in the mountainous regions of southwest China was selected as the research object, and by establishing a multi-indicator evaluation system using a landscape pattern index, a multifunctional identification model, a coupled coordination model, and a geodetector model, the spatial variability in the evolutionary characteristics and the coupling and coordination of cropland landscape ecological security (CLES) and cropland multifunctionality (CM) in the mountainous regions of the southwest and their driving mechanisms were explored. The main results were as follows: (1) CLES in the mountainous areas of southwest China has undergone an evolutionary process of first declining and then slowly rising, with the characteristics of “fast declining in the high-value areas and slow rising in the low-value areas”, while CM showed a spatial distribution of “high in the northwest and low in the northeast”, with positive contributions originating from ecological functions. (2) Over the 20 years, the cropland coupling coordination degree (CCCD) values showed significant spatial heterogeneity, which was regionally expressed as ejective folds (EF) > TF (tight folds) > TLF (trough-like folds) > AF (anticlinorium folds). Low CCCD values were primarily found in the east, whereas high levels were primarily found in the west, with a rapidly diminishing trend. (3) There were differences in the driving mechanisms of CCDD in different landscapes, but GDP was still the determining factor and had a limiting effect. Hence, we call for the adoption of a “function over pattern” approach in areas with more development constraints and a “pattern over function” approach in areas with fewer development constraints. Ultimately, this study will contribute to the formation of a coupled cropland mechanism system described as the “multi-mechanisms drive, multi-elements integrated” system. In conclusion, this study can provide a better understanding of the relationship between cropland patterns and multifunctionality, which can help provide a basis for cropland conservation and landscape planning in similar mountainous areas and promote the achievement of sustainable agricultural development goals in the mountainous areas of southwest China.

## 1. Introduction

With the advancement of high-quality socio-economic development, the need for cropland is no longer restricted to a single function of food supply [1,2], but it is rapidly expanding to include derived purposes such as livelihood security, landscape aesthetics, recreation, and tourism [3,4]. At the same time, due to the pressures of global change and the intensification of human activities, cropland resources across the world continue to face non-agriculturalization, non-food marginalization, and reverse ecology [5,6,7]; in turn, this is constantly changing the cropland landscape patterns [8,9], such as the trend of cropland fragmentation due to the interference of various non-farm activities, which has a significant impact on the continuous supply of multifunctional cropland and limits the sustainable use of cropland resources [10,11]. Therefore, it is important to enhance the understanding of the relationship between landscape patterns and multifunctional evolution in cropland systems to further serve the management of cropland resources.

The key to reducing agricultural landscape degradation and boosting the ecology and economy is improved structural planning and design to adapt to natural conditions and urban–rural interactions [12,13]. The landscape pattern represents the ecological environment caused by human activities and natural elements [14]. It may indicate a region’s ecological environment status [15]. With the rapid change from traditional agriculture to modern agriculture in recent years [16], cropland landscape patterns have shifted from productive to ecological–economic and ecological-regulation-oriented as a result of social ecology feedback [17], and cropland-related research has gradually expanded to assess cropland ecosystem health, landscape ecological security, and ecosystem sustainability [18,19]. Among them, cropland landscape ecological security (CLES) is used to understand the ecological consequences of regional cropland from the perspective of landscape pattern changes, primarily by describing the characteristics of changes in cropland use patterns and assessing the state of CLES under natural and anthropogenic disturbances [20], which can reflect the spatial structure characteristics of cropland uses compatible with economic development [21] and is the best perspective to explore the evolutionary mechanisms and processes of human–nature coupling [22]. However, as a natural–human complex system [23], the cropland system continually carries out material circulation, energy flow, and information transmission processes within the system through the cooperative activity of nature and humans [24]. As a result, the impacts of other land types on cropland ecosystems should be reflected in the index system when assessing the CLES, and quantifying only cropland landscape pattern characteristics will result in imprecise research results, which have been easily ignored in previous studies. Furthermore, as a result of land use changes [25], landscape patterns can influence ecosystem functions by changing the surface properties [26,27], influencing the availability of ecosystem multifunctionality [28,29].

CM is an extension of and a concrete way to achieve ecosystem multifunctionality [30], which has become one of the research objectives that is widely focused on by scholars as a development direction for resource conservation [31]. Previous studies have been carried out on a large number of areas such as the connotations [32], planning and management [33], influencing factors [34], and trade-off synergy [35,36] of cropland multifunctionality. Among them, the multifunctional trade-off synergy relationship of cropland has received high attention [37], which mainly draws on the analytical framework of ecosystem service trade-off synergy studies. These advances have played an important role in the conservation and management of cropland. In recent years, landscape patterns and ecosystem services have become a new focus [38]. Some researchers have observed that landscape fragmentation negatively affects ecosystem service provision [39], potentially reducing the level of human well-being [40]; others have analyzed the coupled coordination of landscape ecological risk and ecosystem services [41,42]. Although research on landscape patterns and ecosystem services has achieved some results, problems still exist. First, as key ecosystems supporting human livelihoods and well-being [43], cropland landscapes have received insufficient attention targeting their landscape ecological security and multifunctionality coupling relationships, with most previous studies being single-sided. Secondly, there is still room for improvement in exploring mechanisms regarding CCCD. Therefore, a coupled research case linking CLES and CM is urgently needed to elaborate more deeply on the evolution path of cropland use in the context of ecological civilization construction and diversified demands and to provide a reference for sustainable and operable land management models.

China has a large population and relatively scarce land resources per capita. Food security has long been a focus of international interest. As an important reserve resource [44], the conservation and development of mountainous cropland require special attention from the government and academia [45]. Sloping cropland, as a critical component of mountainous karst cropland in southwest China, faces issues such as soil depletion, diminishing land productivity, and frequent ecological concerns [46]. Since 1999, when China began its reforestation program [47], it has effectively reduced the area of sloping cropland and stone desertification, leading to significant ecological improvements in recent years in the karst mountains of southwest China [48,49], but the space for agricultural production has also been rapidly decreasing, weakening the economic resources of mountain residents [50]. Weakening the source of income for mountain residents and reduced food production due to excessive greening will inevitably force mountain farmers into the poverty trap [51]. To some extent, this has increased the burden of the population carriers in urban areas and production pressure in primary food production areas [52,53], indicating that current ecological restoration programs must incorporate corresponding social goals in addition to improving the quality of the ecological environment [54], which reflects the necessity for CM management. Therefore, this study selects the karst trough valley area in southwest China as a research case to explore the evolutionary characteristics of the coupling between ecological safety patterns and multifunctionality in cropland landscapes and their driving mechanisms, and to ask the following questions: How does the coupling coordination between ecological safety and multifunctionality in cropland landscapes evolve in the context of environmental protection and the diversity of human needs? Is there spatial heterogeneity and what are the differences in their respective influencing factors? This has important implications for the rational use of cropland resources and the promotion of optimal land resource allocation.

The karst troughs in southwest China are developed on the back slopes, and morphologically the bottoms of the troughs are flat, the accumulation of soil is thick, the distribution is long, the section is “U” shaped, and the longitudinal extension is trough-shaped. This is both an important ecological barrier and ecologically fragile area in southern China [48], but also an area where poor counties are concentrated, and it is a typical area combining “protection” and “development” [55]. The landscape pattern and multifunctionality of the cropland in this region have changed dramatically under different development goals and ecologically oriented policy scenarios, making it a typical representative of cropland use research in the new era.

There is an urgent need to research the coupled evolution of ecological security and the multifunctionality of cropland landscapes in this region, and to propose new ideas for the management of cropland in this region to properly deal with the conflict between resource scarcity and the growth of social demands. Based on this, the main research objectives of this paper include: (1) analyzing the evolution characteristics of cropland under the framework of coupled landscape ecological security and multifunctionality; (2) distinguishing the spatial heterogeneity of the influence of factors on CCCD; (3) exploring the CCCD in different stages of agricultural development; (4) proposing dynamic conservation strategies for cropland landscape systems at an advanced stage. The research framework and results can complement and improve the existing research on the transition of cropland use and provide a reference for cropland conservation policies and the sustainable development of cropland in the new era.

## 2. Study Area, Data Sources, and Framework

### 2.1. Study Area

**Figure 1:**
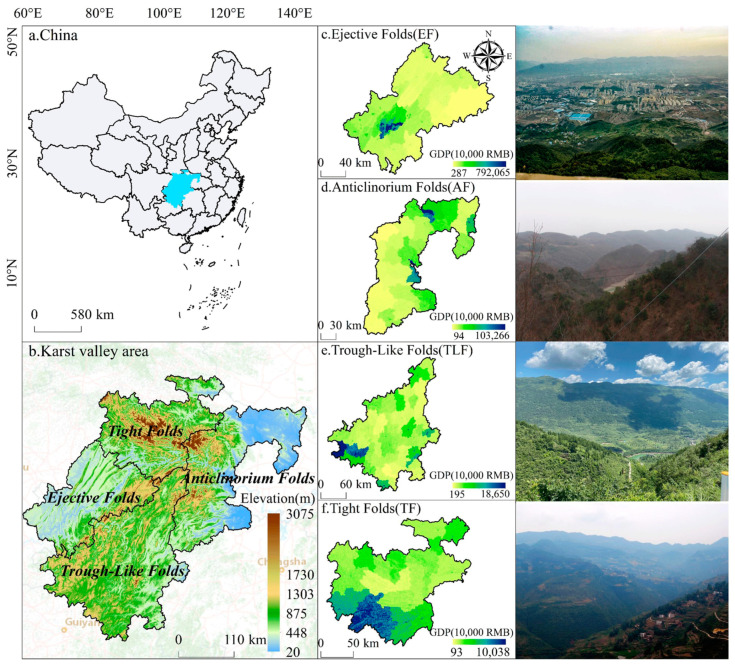
The study area (c-f represents the spatial distribution of GDP under the 1 km ×1 km raster of the karst trough valley landform for four types of ejective folds (EF), anticlinorium folds (AF), trough-like folds (TLF), and tight folds (TF), respectively).

The study area is located in the southwestern part of China. It includes 136 counties in northeastern Guizhou Province, western Hunan Province, western Hubei Province, southeastern Chongqing Municipality, central Chongqing Municipality, and northeastern Chongqing Municipality, China (Figure 1). According to the type of fold assemblages formed by trough and valley landforms [56,57,58], it is composed of four geomorphic areas: tight folds (TF), trough-like folds (TLF), ejective folds (EF), and anticlinorium folds (AF). The GDP performance ranking in 2019 was EF > AF > TLF > TF, with significant socio-economic gradient differences. The total area is 299,900 km^2^, the average annual temperature range is 14–18 °C, the average annual rainfall range is 800~1600 mm, the average altitude range is 500–2500 m, and the humid and rainy sub-tropical monsoon climate dominates the area. Geologically, it shows the distribution of carbonate rocks and clastic rocks. The geomorphology shows the parallel distribution of northeast-oriented monopoly barren striped mountains and trough valleys or long depressions. It is located in the Wuling Mountains of China, where poverty is a prominent problem. It is a typical ecologically fragile area with a complex and diverse natural environment, rich geographical types, and a remarkable geological history, making it a significant ecological zone. The Chinese government has implemented a series of ecological restoration projects in the study area, starting from “returning farmland to the forest” around 1999, “stone desertification control” since 2008, natural forest protection and public welfare forest protection, and karst ecological protection and restoration. The ecological protection and restoration of karst resources have achieved phased results. Undoubtedly, the rapid social development and ecological environment changes have caused great changes in the structure and function of cropland, and the conflict between man and nature is gradually intensifying. The scarce cropland resources and unique geomorphological features of the study area are ideal for identifying the landscape characteristics of cropland areas in different regions, which is extremely important for regulating the transformation of cropland use according to local conditions.

### 2.2. Data Sources

The data used in this study mainly include land use data, statistical data, soil data, topographic data, river data, and traffic network data. In order to facilitate the subsequent exploration of the influencing factors of the coupling coordination of cropland, we have divided some of the above data attributes into four categories, which are the natural background, hydrothermal vegetation, neighborhood, and socio-economic environment (see Table 1). For more details, see the Appendix D. All spatial data are converted to the same coordinates and spatial resolution (1 km × 1 km) to facilitate data processing.

### 2.3. Research Framework

**Figure 2:**
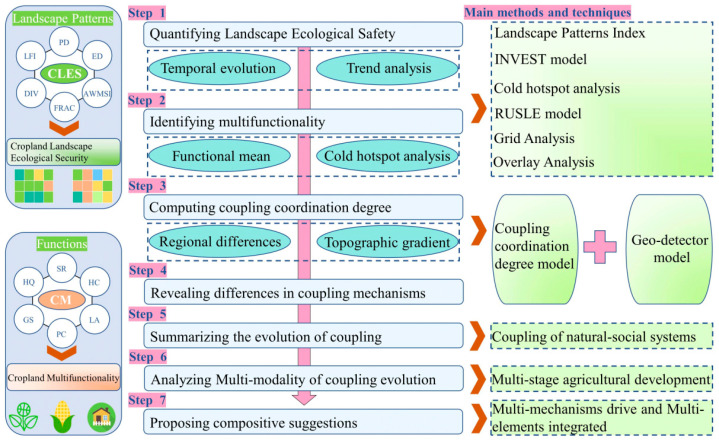
A flowchart used for analyzing the relationship between CLES and CM.

The coupling relationship between the CLES and CM is a phenomenon in which the elements of 2 systems influence each other through interactions. Generally speaking, the layout, size, and shape of cropland and other characteristics provide the carrying role for the CM. In contrast, the promotion and selection of multifunctionality provide a clear direction for reconfiguring the cropland landscape pattern. The coordinated development of the two promotes the healthy development of the cropland use system. Based on the above theoretical understanding, we constructed a research framework for the coupling coordination of CLES and CM (Figure 2). First, a modified landscape pattern ecological security model was used to determine CLES. Second, the cold and hot spot analysis method was used to assess the CM. Third, based on the coupling coordination degree model, the CCCD was calculated. Fourth, the regional variability of the coupling mechanism between CLES and CM was revealed. In addition, a trend surface analysis and topographic gradient were used to characterize the temporal variation and spatial distribution of CLES and CCCD values, respectively. Finally, in revealing the coupling mechanism, we were focused on the goal of coordinated regional development. We, thus, derived the dynamic evolutionary upward model of the “multi-mechanisms drive, multi-elements integrated” system for sustainable cropland development.

## 3. Methods

### 3.1. Constructing the CLES Index

Based on the previous research results [59,60], this study calculated CLES by integrating landscape fragmentation (edge fragmentation and patch fragmentation) and landscape vulnerability values and introduced ecological service values to measure the environmental effects caused by various types of disturbances [61], which not only measured the vulnerability of the cropland ecosystem itself but also truly reflected the dynamic process of the response of the cropland landscape ecosystem to its surrounding environment. A 1 km × 1 km grid was used as the study unit to obtain a spatially accurate expression of the CLES. Meanwhile, we used the extreme difference standard method to standardize the calculation results to between 0 and 1, as this can make the evaluation results comparable. Drawing on the existing research division method [60], the natural interruption method was used to classify the regional CLES into five classes—low, sub-low, medium, sub-high, and high—and the specific calculation formula can be viewed in references [60].

### 3.2. Assessment of Cropland Functions

Functional classification has an important impact on the identification of multifunctionality. In this study, cropland was used as the research object, and concerning more than 20 common landscape functions at present [62], six categories of representative landscape functions were selected for spatial convenience, namely soil retention (SR), habitat quality (HQ), habitat connection (HC), grain supply (GS), population-carrying capacity (PC), and landscape aesthetics (LA).

In general, sloping cropland especially has SR capacity, and the study area is dominated by sloping cropland. Therefore, it is necessary to identify the SR capacity of cropland, which is the difference between potential erosion and actual erosion [63]. The identification results can play an important reference role in soil conservation. The HQ is the potential to provide suitable conditions for species to survive, reproduce and develop, reflecting regional biodiversity [64]. The HC is defined as the ability of a landscape to promote or prevent the ability to move between habitat patches [65], supporting one of the most critical components for the long-term persistence of biodiversity and the maintenance of ecological functions [66]. The GS is the basis of productive human life [67] and is an important type of supply service, given the strong link between agricultural crop volume and net primary productivity [68]. The cropland is an important constraint on the carrying capacity of a country or region’s population [69], and the PC represents the livelihood conditions provided by the agricultural land around a settlement [70]. The LA represent a combination of nature and society [71], and the study of the aesthetics of cropland landscapes leads people to appreciate the aesthetic services that cropland areas provide to enhance human well-being in general [72], in addition to the aesthetic value of the cropland itself, but also in relation to the ease of capturing aesthetic information [73].

The calculation results of each function were standardized and displayed in a 1 km × 1 km grid, with the values ranging from 0 to 1. When the data were processed using the extreme difference standard method, X_max_ and X_min_ denoted the maximum and minimum values of a certain cropland function indicator over all years, respectively, which facilitated the comparative analysis of the spatial and temporal variability of each function and avoided the problem of weak comparability in the calculation results due to the difference in the size of each administrative region. Details and formulae for each cropland function can be found in Appendix A.

### 3.3. Identification of Cropland Multifunctionality

Unlike single functions on land use types at the micro level, multifunctionality has a spatial overlap property, implying that multiple functions may exist simultaneously in a single image element [33]. Since some of the six functions in a single grid did not have clear thresholds, multifunctionality was identified by superimposing the functional hotspots of each cropland area [74] and determining the locations of aggregation of high or low values based on the Z-score. The CM was spatialized to a 1 km × 1 km geographic grid, and the results of the study were more accurately spatially represented compared to previous administrative scales, using the following identification formula:

(1) $$G_{i}^{*}(d)=\sum_{i=1}^{n}W_{ij}(d)X_{i}/\sum_{j=1}^{n}X_{j}$$

(2) $$Z(G_{i}^{*})=\frac{G_{i}^{*}-E(G_{i}^{*})}{\sqrt{Var(G_{i}^{*})}}$$

(3) $$CM_{i}=\sum_{i=1}^{6}\omega_{i}\times CF_{zi}$$

(4) $$CM_{sta}=(CM_{i}-CM_{\min})/(CM_{\max}-CM_{\min})$$

where G_i_* is the clustering index of spatial cell *i*, *Z* is the significance of this clustering index, *W_ij_* is the spatial weight defined in terms of distance, *X_i_* and *X_j_* denote the functional values of grid *i* and grid *j*, respectively, and E (G_i_*) and *Var*(G_i_*)are the mathematical expectation and variance of G_i_*, respectively. *CM_i_* is the total number of hot spots for cropland functions in each grid, *CF_zi_* is the Z-score of cropland functions of category *i*, and *ω_i_* is the weight of each type of cropland function, which is set to 1. *CM_sta_* refers to the normalized value of CM and *CM*_min_ and *CM*_max_ are the minimum and maximum values of CM, respectively. The higher the CM value, the greater the multifunctional capacity of the cropland system.

### 3.4. Coupling Coordination Analysis of CLES and CM

The coupling coordination model in physics measures the coupling coordination degree between the CLES and the CM at time t by calculating the coupling between the two. The calculation formula is:

(5) $$U=2\sqrt{(u_{1}\times u_{2})/{\left(u_{1}+u_{2}\right)}^{2}}$$

(6) $$D=\sqrt{U\times T}$$

(7) $$T=\alpha u_{1}+\beta u_{2}$$

where *U* represents the coupling degree, T is the comprehensive evaluation index between CLES and CM, *D* is the CCCD (0 ≤ *D* ≤ 1), and α and β denote the weight coefficients of the two, respectively. Taking into account the equal importance of the two under the coordinated development, *α* = *β* = 0.5, *u*_1_ represents CLES (0 ≤ *u*_1_ ≤ 1), and *u*_2_ represents CM (0 ≤ *u*_2_ ≤ 1). According to the analysis results for the CCCD, it is divided into the following five classes: severe disorder, mild disorder, intermediate coordination, good coordination, and excellent coordination.

### 3.5. Geodetector

This paper focuses on exploring the mechanisms of interaction between CLES and CM and their regional variability, contributing to a comprehensive understanding of the mechanisms underlying the evolutionary divergence of the CCCD. Concerning the relevant studies of both, 16 representative, easily quantifiable, and accessible impact factors were selected as independent variables and the CCCD was considered as the dependent variable, while their details are shown in Appendix C.

The factor detector was mainly used to detect the spatial heterogeneity of the dependent variable. By calculating the q-value, we can determine the explanatory power of the independent variable on the dependent variable. The following formula calculates the q-value:

(8) $$q=1-\frac{1}{N\sigma^{2}}{\sum_{h=1}^{L}N_{h}\sigma }_{h}^{2}=1-\frac{SSW}{SST}$$

(9) $$SSW=\sum_{h=1}^{L}N_{h}\sigma_{h}^{2},\;SST=N\sigma^{2}$$

where *q* denotes the explanatory power, *q* ∈ [0, 1]; *h* (*h* = 1, 2,…, *L*) denotes stratification variables; *N* and *Nh* are the sample sizes in stratum *h*, respectively; *σ*^2^ and *σ_h_*^2^ are the variances of the dependent variables in the whole region and category *h*, respectively.

## 4. Results

### 4.1. Spatial and Temporal Evolution Characteristics of CLES in Karst Trough Valley Area

**Figure 3:**
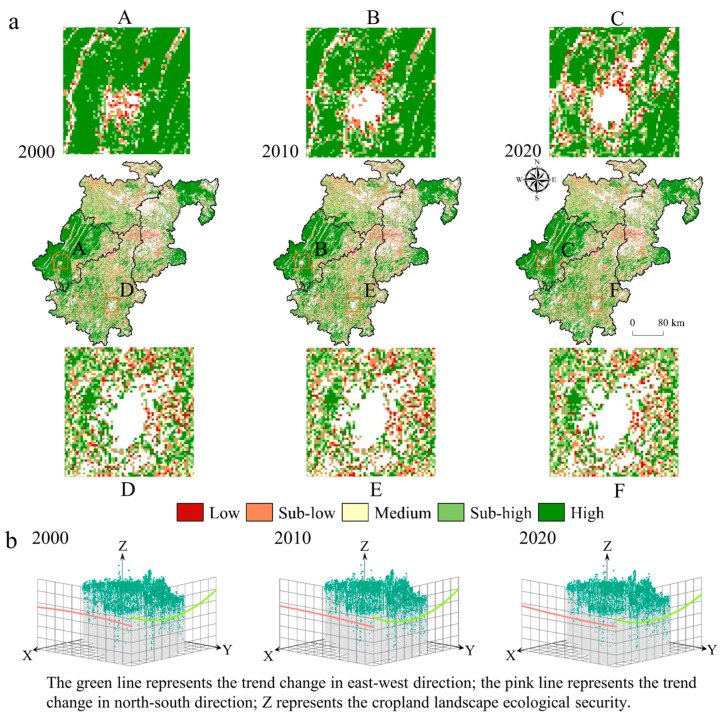
Spatial and temporal distribution of the CLES in karst trough valley areas: (**a**) spatial and temporal distribution characteristics; (**b**) trend analysis.

Calculations based on the Landscape Ecological Safety Index showed that spatially, the higher levels of CLES were distributed in the southwest and northeast. In comparison, the lower classes were distributed primarily in the northwest, with a regional expression ranking of EF > AF > TLF > TF. The average values of CLES in 2000, 2010, and 2020 were 0.6149, 0.6107 and 0.6162, respectively, with an overall decreasing and then increasing trend. From the local areas with large changes, the land use types in the gaps of parts A, B, and C were constructed landscapes (Figure 3a), which showed the impact of human activities on CLES. Specifically, it seems that with the advancement of urbanization, construction land occupies a large amount of cropland. The ability of the cropland system to resist external disturbances decreases sharply, increasing landscape vulnerability, so that the low value of the CLES expands to the outskirts of the city. Still, because the areas near the distribution of human settlements were generally flatter in topography, the scale of cropland within the territory was larger. The patch connectivity is higher, and the areas farther away from the town center have a higher CLES and are also highly susceptible to disturbance. In contrast, the land use types in some D, E, and F gaps are mainly ecological landscapes, which show the disturbance of natural factors on the cropland landscapes. The value and variation of CLES in this region are small, and a “heterogeneous” distribution characterizes the spatial distribution. The trend in the evolution of CLES based on geostatistical trend analysis methods (see Appendix B) (Figure 3b) and the trend of ecological safety of the cropland landscape in 2000 were significant, with an obvious positive “U” shape in the east–west direction, with the highest and lowest values being found in the central part in the west, and a straight line in the north–south direction, with the southern part being higher than the northern part. The low point of the “U” shape is getting lower and lower, indicating that the landscape ecological safety of the cropland system is decreasing. In 2020, the north–south direction was slightly higher in the south and north and slightly lower in the west in the east–west direction.

### 4.2. Temporal Evolution and Spatial Distribution of CM

**Figure 4:**
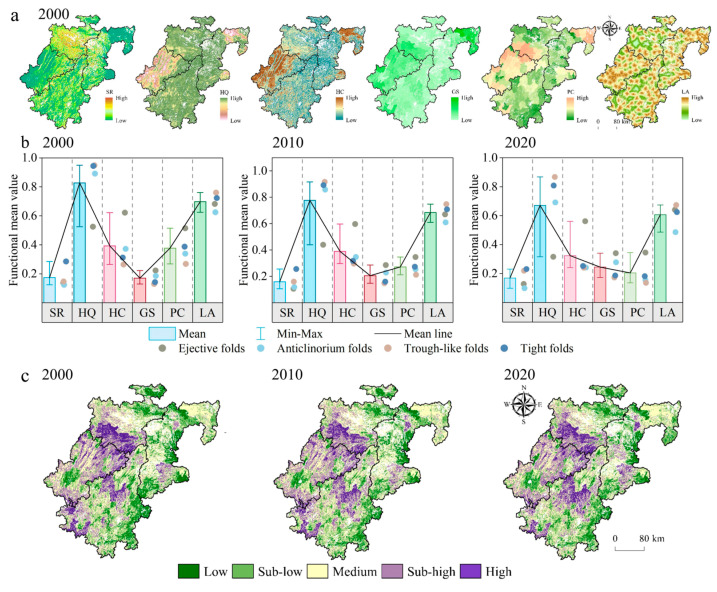
Temporal evolution and spatial distribution of single functions and multifunctionality: (**a**) spatial and temporal distribution characteristics; (**b**) functional mean values; (**c**) cropland multifunctionality; SR: soil retention; HQ: habitat quality; HC: habitat connection; GS: grain supply; PC: population-carrying capacity; LA: landscape aesthetics.

The computational model for each cropland function gives the spatial and temporal distribution and mean values for each function (Figure 4a,b), where in 2000 the mean values for RS, HQ, HC, GS, PC, and LA were 0.17, 0.82, 0.39, 0.17, 0.37, and 0.69 respectively, with high HQ and LA values for the same ecological function and low GS values for the production function. The regional differences in soil retention function are small, with a gradual upward trend in TLF (Figure 4a); the HQ is smaller in EF, while HC and PC are higher (Figure 4a), which is doubly influenced by the high social development level and flat topography of the region, but the PC value decreases significantly due to the increase in urbanization rate, accompanied by a rapid decline in the rural population. The variability in the spatial distribution of GS is gradually increasing, which correlates with the level of regional scientific and technological development. The areas with smaller LA values are the main areas to be avoided due to various policies, mainly in terms of EF, with less undulating terrain, which can effectively control the “de-fooding” and “de-agriculturalization” of cropland, while the higher areas can be considered as potential tourism resources and advantageous areas for the development of ecological agriculture. According to the formula of multifunctionality, one can obtain the spatial distribution of CM (Figure 4c). It has a spatial distribution pattern of “high in the northwest and low in the northeast”, with obvious spatial polarization, showing the evolution of the simultaneous expansion of high and low values. In the urban areas of Shapingba, Jiulongpo, and Yuzhong in the EF, where the infrastructure is well developed and the level of development is high, a distribution pattern of “homogeneous in the central urban areas and radial in the suburban areas” has been formed, while the CM forms in the mountainous areas of Youyang and Pengshui show a geospatial pattern of high-value clustering, indicating that the changes in agricultural development patterns driven by the concepts of high-quality economic development and ecological civilization have led to a heterogeneous evolution of the spatial pattern of multifunctional agriculture.

### 4.3. Coupling Coordination Degree of the CLES and CM

**Figure 5:**
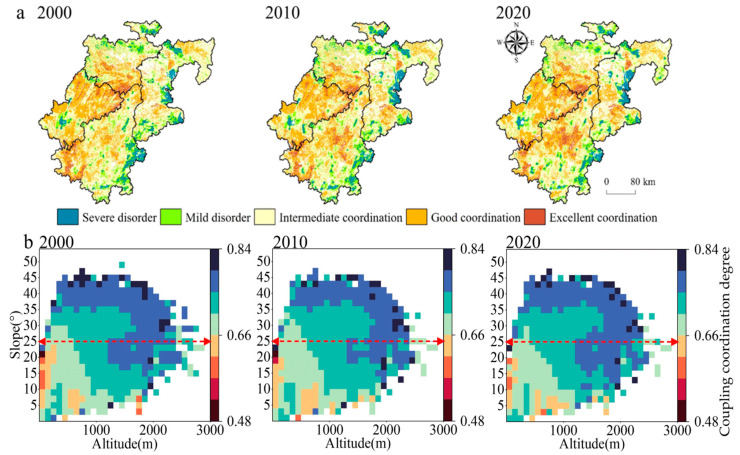
Temporal evolution and spatial distribution of coupling coordination degree values: (**a**) spatial and temporal distribution characteristics; (**b**) topographic gradient characteristics: above the red dashed line arrow in the figure are the distribution characteristics of CCCD of sloping cropland suitable for reforestation, while the below are the distribution characteristics of CCCD of cropland with good production potential.

According to the coupling coordination model, it can be seen that the spatial distribution pattern of the CCCD is high in the west and low in the east, with a clear simultaneous expansion of high- and low-value areas (Figure 5a). For 2000, 2010, and 2020, the mean CCCD values are 0.7108, 0.7025, and 0.7053, respectively, and the overall trend is consistent with the CLES values. Among the CCCD classes, the severe disorder and mild disorder classes are smaller in scale and have a more “heterogeneous” spatial layout; the intermediate coordination class is larger in scale than the above two types. Good coordination is seen mainly in the northern part of the EF, which indicates that the CLES in this region is in a good “coexistence relationship” with CM; the excellent coordination class is on a smaller spatial scale, indicating that the coupled coordination of cropland still existed. The high-coordination areas, which have changed most significantly over the past 20 years, continue to expand outwards, forming new “high-value areas”, while the severe disorder, mild disorder, and intermediate coordination areas are spreading outwards. The tendency for severe disorder, mild disorder, and intermediate coordination areas to spread outwards is also obvious. For instance, in Banan and Yubei, where the level of urbanization development is high, the massive occupation of cropland by urban expansion in recent years has reduced the areas of CM. According to the hotspot mean map of the coupled coordination of cropland areas (Figure 5b), we can see the topographic slope has a positive relationship with the coupled coordination of cropland. In other words, most of the cropland in the study area is sloping, and the development of the region still needs to receive more attention. Below the 25° slope, the regional spatial heterogeneity of the coupled coordination of the cultivated land is obvious and less stable; above the 25° slope, the CCCD has the opposite characteristics. In conclusion, the differences in CCCD values between regions are closely related to social development, the topographic conditions, the intensity of human activities, and cropland resource endowments.

### 4.4. Factor Detection Analysis

**Figure 6:**
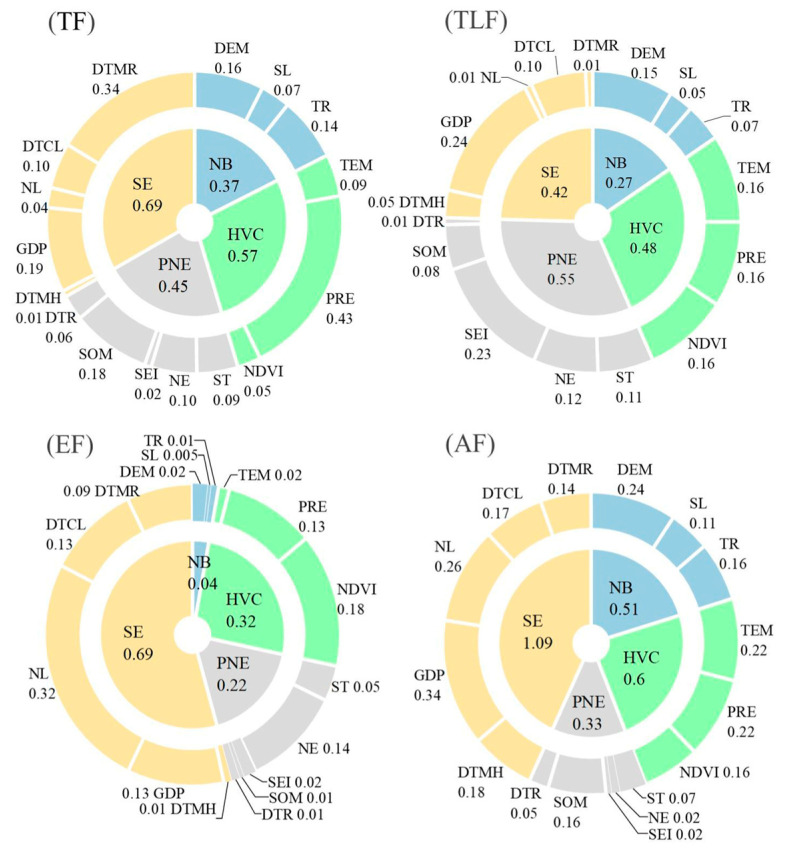
Distribution of q-values of factors influencing different karst trough valley types: NB: natural background; DEM: elevation; SL: slope; TR: topographic relief; SE: social environment; DTMH: distance to main highways; GDP: gross national product; NL: night lights; DTCL: distance to county location; DTMR: distance to main railways; HVC: hydrothermal vegetation condition; ST: soil type; NE: neighborhood enrichment; SEI: soil erosion intensity; SOM: soil organic matter; DTR: distance to rivers; PNE: production neighborhood environment; TEM: annual average temperature; PRE: annual average precipitation; NDVI: normalized difference vegetation index.

The geographic detector was applied to four regions, and 16 factors were selected for 2020 to detect the regional variability of the dominant factors of the CCCD. The results of the factor detector analysis are shown in Figure 6, and all influencing factors were highly significant (*p* < 0.001). The results showed significant differences in the influencing factors of the spatial distribution of the CCCD values, but the GDP and annual average precipitation were still the dominant factors. In TF, the top 5 influencing factors according to q-value statistics are the annual average precipitation, distance to main railways, GDP, soil organic matter, and elevation, which play a decisive role in the spatial distribution pattern of CCCD values, among which the influence of SE is the most significant. In TLF, the explanatory power of the SE factor is still greater than that of the NB factor. The explanatory power of the TEM, PRE, and NDVI in the HVC factor is stronger. The most influential factor in the PNE factor is the soil erosion intensity. In EF, the explanatory power of the SE factor is the highest and more significant than the other three factors. In AF, the explanatory power of the factors in the NB factor increased compared to the other three zones, with the largest explanatory power being the DEM, while the explanatory power levels of the SL and TR are 11% and 16%, respectively. The combined explanatory power of the CCCD values among the four trough types are ranked as SE > HVC > PNE> NB, PNE > HVC > SE > NB, SE > HVC > PNE > NB and SE > HVC > NB > PNE. In summary, the socio-economic factors had a greater influence on the CCCD than natural factors and were not strongly correlated with the spatial location.

## 5. Discussion

### 5.1. The Evolution of CLES with CM of Multiple Types

Conducting research on the ecological safety of cropland landscapes improves land managers’ effective grasp of ecological changes in cropland areas and helps to improve and protect cropland ecosystems. This study differs from the previous studies on large administrative areas or watersheds [75,76], and instead we selected four typical trough and valley landscape types located in the mountainous regions of southwest China at different levels of economic development for a comparative analysis. Significant variability exists between the different security levels (Figure 3). Land managers can designate high-quality cropland areas around central urban areas, which is an effective way to protect peri-urban cropland resources, while cropland areas located near ecological land areas, which were more stable during the study period, can be protected by identifying high-value cropland, although the landscape is relatively fragmented, and this emphasizes the importance of regional biodiversity [77], which is conducive to the sustainable management of cropland in mountainous areas.

The identification of multifunctional spatial patterns of cropland has become a core component of multifunctional research [35,37]. However, as the demand for cropland functions gradually evolves towards diversification [37], the synergies and trade-offs between cropland multifunctionality are intensified. The synergistic relationship between GS, PC, and HC in this study (Figure 4a) is consistent with the findings of Peng et al. [34]. Of these, GS and PC were more highly correlated. Areas with higher cropland connectivity also had higher food supply capacity, which is also consistent with the actual situation; GS and HQ showed a trade-off relationship, as it is known that large ecological restoration projects improved the cropland ecosystem regulation function, but it was also found that the trade-off between the cropland supply function and regulation function is becoming more and more obvious [78], which requires the effective performance of the multifunctional management model for the sustainable development of the cropland. This requires the effective use of multifunctional management models as the core of the sustainable development approach. Therefore, further research on multifunctional trade-offs and synergistic relationships of cropland areas at multiple scales from multiple regions can be carried out in the future to provide detailed information to land stakeholders at different levels.

**Figure 7:**
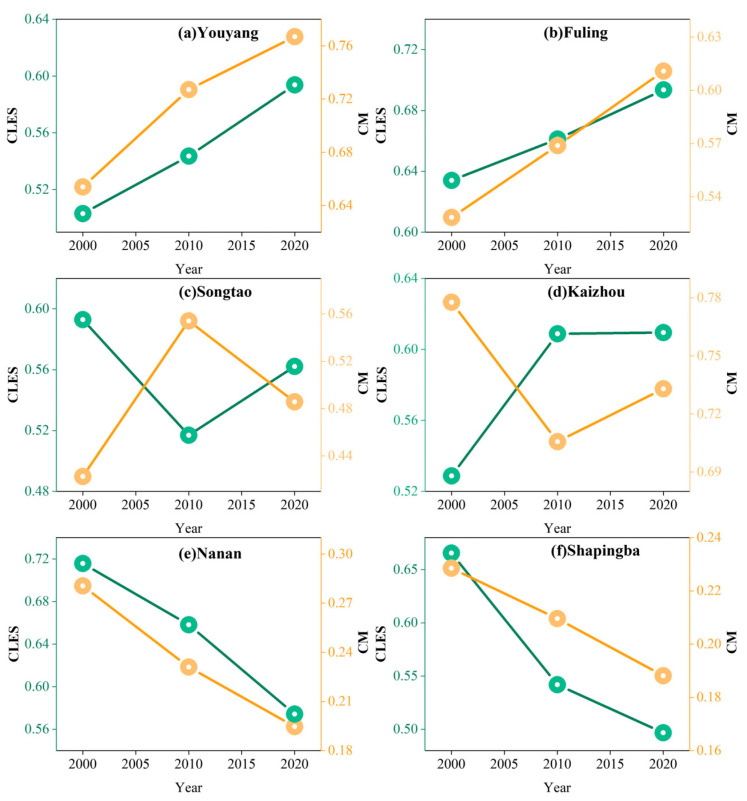
Multistage representation of CLES and CM evolution of multiple types: CLES: cropland landscape ecological security; CM: cropland multifunctionality.

The coupled system of landscape patterns and multifunctionality in cropland is in a dynamic evolution process, influenced by the diversification of human needs and the continuous development of society. Combining the evolution types of both can help effectively predict and control the evolution direction of cropland and help to develop more scientific strategies for optimizing land management and human activities. Consequently, we selected six typical counties, which can be divided into three typical types of interaction changes—mixed, mutual inhibition, and mutual facilitation types—corresponding to the mechanisms of multiple stages of agricultural development. The first type is at the early stage of modern agricultural development, where human subsistence needs influence the cropland system. The elements, mechanisms, and order within the system are more disordered. CLES and CM are dominated by mixed types (Figure 7c,d), such as in Songtao and Kaizhou. The agricultural economy usually dominates this stage, and the state carries out large-scale cropland development work. As a result, the cropland area increases significantly and the reserve resources of the cropland become more abundant. When the impacts of human activities on cropland ecosystems are weak, the negative effects of cropland expansion can be absorbed and offset to a certain extent, as shown by the increase in CLES and the decrease in CM; conversely, the changes in both are opposite. The second type is in the middle stage of modern agricultural development, whereby multiple mechanisms limit the cropland system. The CLES and CM mostly show mutual inhibition types (Figure 7e,f), such as in Nanan and Shapingba. Most counties in this period sacrifice the ecological environment for socio-economic development. The rapid loss of cropland resources under urban construction, industrial land expansion, non-crop cultivation, and other demands show an increasingly severe trend at this stage. Meanwhile, this also means that the intensity of human activities intensifies and the area, shape, space, and other various characteristics of the cropland landscape change, leading to changes in the ecological environment from single to complex and from continuous to discontinuous, while the health of the regional cropland ecosystem is threatened. The two are negatively constrained and in a negative feedback state; the third type is the late stage of modern agricultural development, which is mainly characterized by the growing scale of industrialized operations, the increasing level of mechanization, and the rapid rise of modern leisure agriculture. Multiple mechanisms drive the cropland system, and CLES and CM are almost characterized by mutual facilitation (Figure 7a,b), as in Youyang and Fuling. The two complement each other, forming a virtuous promotion and positive feedback system, providing the basis for the deep integration of “ecology and industry.” At this stage, with the help of land management policies, the development of urbanization and new rural construction puts forward higher requirements for the utilization of scarce cropland resources, which leads to the intensive and economical utilization of cropland, the reconstruction of the spatial pattern of land use, the protection of cropland ecosystems, and the improvement of agricultural technology, bringing into play the multifunctionality of cropland areas, whereby the phenomenon of the construction occupation of cropland areas is moderated and the loss rate of cropland areas gradually decreases.

### 5.2. Taking Regional Variability and Multiple Factors into Account in Cropland Landscape Management

**Figure 8:**
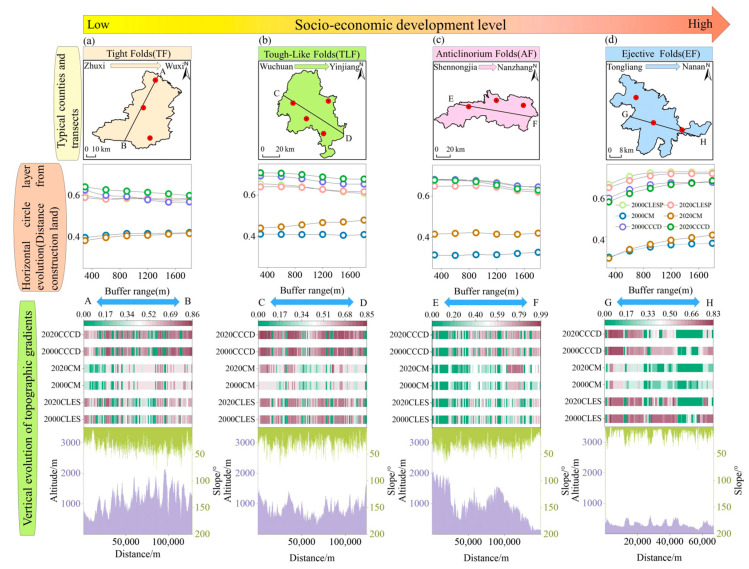
The evolution of vertical in topographic gradient and horizontal circle layer distance from construction land in typical counties in CLES, CM and CCCD: (**a**) in TF; (**b**) in TLF; (**c**) in AF; (**d**) in EF; CLES: cropland landscape ecological security; CM: cropland multifunctionality; CCCD: cropland coupling coordination degree.

The spatial differentiation of the cropland coupling coordination degree resulted from multiple factors of the natural environment and socio-economic conditions, and was a complex process. Cropland landscape planning and management initiatives must consider the spatial correlation between natural and socio-economic processes and their interaction in the landscape. Rapid social development accelerates the market value of cropland, induces the restructuring of agricultural production, leads to changes in the structure of cropland use, and drives the coupled evolution of the spatial pattern and functions in cropland areas. In regions with the high–low coupling of socio-economic development levels and topographic gradients, for such as in Tongliang and Nanan in EF (Figure 8a), this region has higher regularity, a higher scale of cropland patches, and higher spatial and multifunctional coupling coordination. The CLES and CM coupling relationship is a significant “urban–peri-urban” gradual spatial transition. They show a “slowly increasing–stabilizing” trend. This evolution process appears in the distance value and can be used for land management. It can be used as a threshold reference for land management and can play a role in balancing cropland protection and urban spatial expansion interests. However, the spatial layout of the cropland in the low–high coupling areas of the socio-economic development level and topographic gradient, such as in Wuchuan to Yinjiang in TLF (Figure 8b), are more dispersed, with a smaller patch area and lower spatial and functional coupling in the cropland. Among them, the further away we get from the construction land, there is a gradual decrease in the CLES, which is related to the decline in the spatial distribution of the cropland, while there is a rising trend in the combination of multifunctional cropland extremes, which is a response to the gradual increase in the ecological functions of cropland under the influence of environmental engineering policies. However, in most regions the development of CM lags behind CLES (Figure 8), so there is still room to improve the double-high model of both factors.

In summary, the empirical results for the karst trough valley region show that the various factors influence the CCCD, including changes in the intensity of human activity, the geographic location, and the socio-economic development conditions. The geodetector model allows us to see the regional variability of the influencing factors (Figure 6), which highlights the need for future regional development to grasp the multifactor drive for coupled cropland patterns and functions. There is a need to improve the ability of land managers to solve problems in an integrated manner to face the impacts of diverse mechanisms on cropland systems, and ultimately to guarantee the route of the virtuous ecological–economic–social cycle development in mountainous areas.

### 5.3. Contributions and Policy Implications

Currently, in the context of rural revitalization and ecological conservation, cropland is involved in human production activities as an important factor [50], and human activities change the landscape patterns of cropland use through different land use strategies, which in turn change the ecological processes and ultimately affect the supply of cropland functions. The framework proposed in this study is appropriate for this case and can be extended to other study areas or land-type studies. For the foreseeable future, global cropland development, driven by an expanding human footprint and food security, can lead to increased production capacity [38] but will affect farmland ecosystems, and cropland management models urgently need to adapt to the changing environment. We hope this study will inform others on how cropland use is controlled and policy is implemented, contributing to a dynamic spiral of evolutionary paradigms that will ultimately seek to contribute positively to SDG2 and SDG12. In particular, as our study area is a typical mountainous area combining urban and rural areas, the conclusions are more extensible and applicable, especially for the goal of achieving integrated urban–rural development, which is a requirement for building a new type of urban–rural relationship in the new era. To a certain extent, this study can provide guidance for solving the conflict between the ecological environment and food security and can provide a reference for harmonizing regional human–land relations.

**Figure 9:**
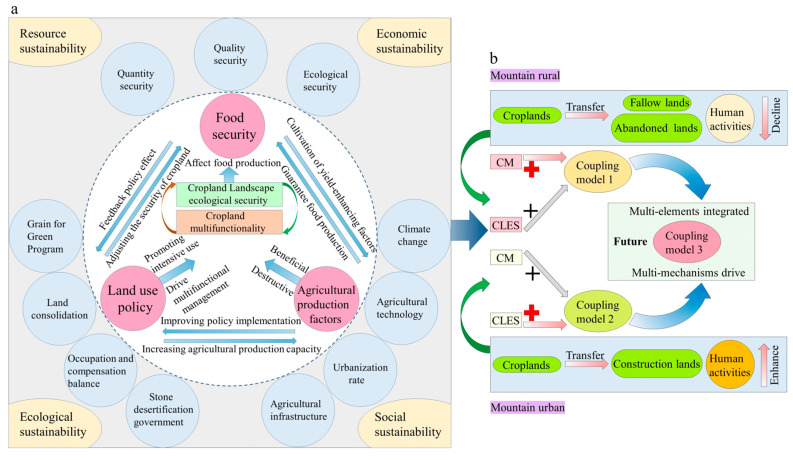
The advanced stage development model for the sustainable management of cropland: (**a**) driving mechanisms for CLES and CM coupled systems; (**b**) the future of coupled models of sustainable development of cropland; CLES: cropland landscape ecological security; CM: cropland multifunctionality.

The study results show that the CCCD is higher in regions with large topographic gradients, such as Changyang County, which are usually endowed with superior natural resources. The quality of cropland resources is poor, and the level of fragmentation is high. These regions should cultivate an integrated model of the ecotourism industry to exploit the functional value of the cropland resources fully. The CCCD in areas with smaller topographic gradients is low, such as in Jiangbei District, which is the core zone of economic growth, with the foundation and potential for high-tech development and more space for cropland multifunctionality enhancements. Among others, the goal of mountainous cropland management is to break through the natural factors caused by the primary “small scale, broken patches” limit, but technically this is difficult. However, it should be emphasized that this region’s cropland has multiple ecological attributes. The focus is on demonstrating the climatic regulation of the cropland system, as well as water conservation, biodiversity conservation, recreational recreation, and other ecological landscape functions. This approach should be introduced to improve the function over pattern pathway, as in the coupling model 1 (Figure 9b). In contrast, the cropland ecosystem in the plain is easily disturbed by the outside world, the stability is poor, and the conflict between humans and the land is prominent. Regardless, the higher level of social development in the region will stimulate the growth of CM. The focus of cropland remediation initiatives is to strengthen the ecological bottom-line constraints, strictly control the intensity of land development and utilization, and improve the ecological and environmental management processes to maintain and enhance the balance of the entire regional ecosystem. This approach should be introduced to improve the pattern over function pathway, as in coupling model 2 (Figure 9b). The next round of active promotion of land use policies and improvements of agricultural production factors will drive the demand for a new round of comprehensive cropland management initiatives. Relevant policymakers should work on the premise that the existing ecological benefits will not be reduced and should then fully consider the spatial pattern and multifunctional coordinated development to ultimately enhance the public interest in food security (Figure 9a). Based on the above, we deduced the dynamic coupling ascent model as the future direction of agricultural transformation, which is required to integrate the characteristics of plains and mountains, leading to the “multi-mechanisms drive, multi-elements integrated” coupling mechanism system in coupling model 3 (Figure 9b), which can also coordinate the integrated social–natural–economic resource effects and strengthen the endogenous dynamics of a sustainable cycle within the cropland system, which will be conducive to the balanced development of regional cropland spatial patterns and multifunctionality, thereby achieving significant results in terms of regional sustainable development.

### 5.4. Limitations and Prospects

This paper assesses the spatial and temporal evolutionary characteristics of the coupling coordination of CLES and CM, contributing to a deeper understanding of sustainable transition patterns of cropland. However, our study has some limitations and uncertainties. First of all, it is necessary to re-state that the trough and valley landforms in this paper are divided according to the fold combination pattern of trough and valley formations, only to facilitate the realization of the purpose of this paper without reference to precise classification criteria. Regardless, this does not affect the results of the study in essence. We did not use high-resolution data and fieldwork data. In addition, in terms of quantifying the influencing factors, our selection of influencing factors was not comprehensive enough, and the selection of other factors, such as the fertilizer use, pesticide use, and cash crop yield, should be considered in the future. In the next step, we will use high-precision models and data to study the transition of cropland uses from the microscopic perspectives of villages and farmers and use prediction models to predict the evolution of both, scientifically explore the reciprocal “pattern–function” feedback in different scenarios and scales, and strengthen the study of pattern–function–human well-being interrelationships in landscape ecological safety. In addition, we will enhance the research on the landscape ecological safety pattern–function–human well-being interrelationships to provide better land resource management recommendations.

## 6. Conclusions

This study was based on the theory of cropland use transition, and we constructed a research framework of cropland use evolution from a coupled perspective by connecting landscape ecological security and multifunctionality patterns, then applied this conceptual framework to the study of the cropland evolution in the karst trough valley area of southwest China, in an attempt to innovate management ideas for cropland areas driven by rapid ecological restoration. The main findings of this study were as follows.

(1) The CLES experienced a process of decreasing and then increasing, with the high values mainly distributed in the southwest and northeast, while the low values were mainly distributed in the northwest, regionally expressed as EF > AF > TLF > TF.

(2) From the perspective of individual functions, the HQ and LA, which are also ecological functions, were high; the GS, a production function, was low; the regional differences in SR were small; and the HC and PC gradually decreased.

(3) The regional variability in CM is obvious, and it shows a spatial distribution pattern of “high in the northwest and low in the northeast”. The phenomenon of spatial polarization is obvious, showing the evolution characteristics of the simultaneous expansion of high and low values.

(4) Socio-economic development has a suppressive effect on the enhancement of the CCCD, which is most pronounced in the EF region.

In general, areas at high altitude have a diverse mix of cropland functions and mostly exhibit high cropland ecological functions. Usually, the ecological security pattern of the cropland landscape in such regions is more dangerous; therefore, enhancing the multifunctionality of cropland areas by optimizing the cropland patterns in the mountainous regions of southwest China is a key step in addressing the sustainable use of cropland resources. By collating the dynamics data for the coupled evolution of cropland areas, and based on the goal of coordinated regional development, we derived a dynamic coupled upward model of the “multi-mechanisms drive, multi-elements integrated” system for the coming period. These findings will help respond to the goal of integrated urban–rural development; cope with ecological degradation, soil erosion, and land degradation; and regulate non-agricultural disturbances caused by various human activities.

## Figures and Tables

**Table 1:** Data sources and descriptions.

Indicator Types	Data Names and Abbreviations	Data Sources	Spatial Resolution
Natural background (NB)	Elevation (DEM)	Geospatial data cloud (http://www.gscloud.cn/ (accessed on 12 February 2022))	30 m
	Slope (SL)	Calculated from DEM data	
	Topographic relief (TR)		
Hydrothermal vegetation Conditions (HVC)	Annual average precipitation (PRE)	Resource and environment data cloud platform (http://www.resdc.cn/ (accessed on 15 February 2022))	1 km
	Annual average temperature (TEM)		
	Normalized difference vegetation index (NDVI)	United States Geological Survey (http://lpdaac.usgs.gov (accessed on 15 February 2022))	
Production Neighborhood environment (PNE)	Neighborhood enrichment (NE)	Calculated from LULC data	30 m
	Soil type (ST)	Harmonized World Soil Database (http://westdc.westgis.ac.cn/ (accessed on 18 February 2022))	1 km
	Soil organic matter (SOM)		
	Soil erosion intensity (SEI)	Resource and environment science and data center (http://www.resdc.cn (accessed on 18 February 2022))	
	Distance to rivers (DTR)	National Earth System Science Data Center (http://www.geodata.cn (accessed on 18 February 2022))	1:4,000,000
Socio-economic environment (SE)	Gross national product (GDP)	Resource and Environment Science and Data Center http://www.resdc.cn (accessed on 1 June 2022)	1 km
	Distance to country location (DTCL)	National Geomatics Center of China (http://www.webmap.cn/ (accessed on 5 January 2022))	1:4,000,000
	Distance to main highways (DTMH)		
	Distance to main railways (DTMR)		
	Night lights (NL)	National Oceanic and Atmospheric Administration (http://www.ngdc.noaa.gov/dmsp (accessed on 18 February 2022))	1 km
-	Net Primary Production	United States Geological Survey http://www.usgs.gov/ (accessed on 15 February 2022)	500 m
-	LULC data	Resource and Environment Science and Data Center http://www.resdc.cn/ (accessed on 20 February 2022)	30 m

**Figure A1:**
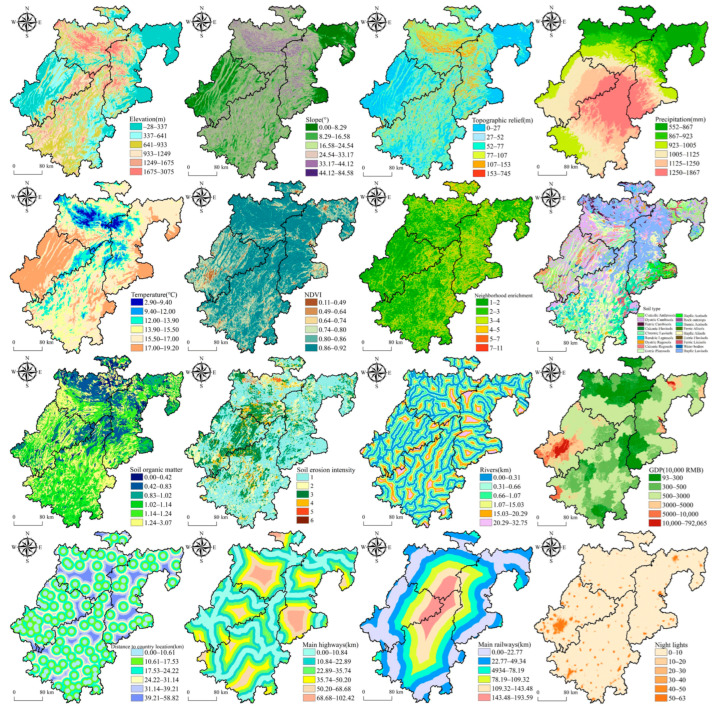
Influencing factors in the study area. Note: The soil erosion intensity scale is divided into six levels: slight erosion (1); mild erosion (2); moderate erosion (3); strength erosion (4); extreme strength erosion (5), intense erosion (6).

## Data Availability

Not applicable.

## Appendix A. Assessment of Cropland Functions

### Appendix A.1. Soil Retention

Generally speaking, sloping cropland has soil conservation capacity, and it is necessary to identify the soil conservation function of the cropland in the study area, which is dominated by sloping cropland. This study used the commonly used modified universal soil loss equation (RUSLE) model to evaluate the soil conservation function [79]. The rainfall erosion force was calculated from the precipitation data, the soil erodibility was defined based on soil texture parameters from the World Soil Database, and the topographic factors were calculated from the DEM for the slope length. The vegetation cover was inverted using the NDVI. In addition, since this study did not separate the management approaches for different types of farmland, the management factor can be considered as a constant value and excluded in the standardization process, calculated as:

(A1) A=R×K×LS×(1−C×P)

where *A* is the soil conservation amount, *R* is the rainfall and runoff factor, *K* is the soil erodibility factor, *LS* is the slope length factor, *C* is the surface vegetation cover factor, and *P* is the soil and water conservation measure factor, referring to the study in the karst areas.

### Appendix A.2. Habitat Quality

The habitat quality function of cropland refers to the potential of cropland ecosystems to provide suitable conditions for species to survive, reproduce, and thrive, reflecting regional biodiversity. This study quantifies the habitat quality of cropland using the habitat quality sub-model of the InVEST model. All building sites were considered threat sources in this paper. The spatial attenuation distance range was set to 4–10 km according to the maximum spatial impact range of towns and cities and the small impact range of other construction land areas with reference to previous research results. The habitat quality index was calculated as follows [80].

(A2) $$Q_{xj}=H_{j}\times \left(1-\frac{D_{xj}^{z}}{D_{xj}^{z}+K^{z}}\right)$$

where *Q_xj_* is the habitat quality of raster *x* in land use type *j*; *H_j_* is the habitat suitability of land use *j*; *D_xj_* represents the degree of habitat degradation; *z* and *k* are scaling factors (constants).

### Appendix A.3. Habitat Connection

Habitat connectivity is defined as the ability of a landscape to facilitate or prevent movement between habitat patches, supporting one of the most critical components for the long-term persistence of biodiversity and the maintenance of ecological functions, so landscape connectivity was added to the multifunctional evaluation index system when assessing the multifunctionality of cropland. In this paper, with reference to the results of previous studies, the landscape importance index calculation method was used. According to the formula, the change in total connectivity after removing a patch is taken as the importance of that patch. Therefore, the larger the area of a patch and the more nodes it has, the higher its connectivity importance, which is calculated using the following formula [81]:

(A3) $$IIC=\frac{\sum_{i=1}^{n}\sum_{j=1}^{n}\frac{a_{i}\cdot a_{j}}{1+nl_{ij}}}{A_{L}^{2}}$$

(A4) $$dI(\%)=100\frac{IIC-IIC_{\mathrm{remove}}}{IIC}$$

where *dI* (%) denotes the contribution of individual patches to the overall connectivity; *IIC*, *IIC* remove is the remaining patch connectivity after removing individual patches; *n* denotes the total number of nodes in the landscape; *a_i_* and *a_j_* denote the areas of patch *i* and patch *j*, respectively; *nl_ij_* denotes the number of connections between patch *i* and patch *j*; AL is the area of the landscape.

### Appendix A.4. Grain Supply

The grain supply is an important supply service that underpins human production and livelihoods. Given the close link between the agricultural crop volume and net primary productivity (NPP) [82], in this paper we corrected the food production of the raster by NPP to achieve a spatial representation of the food supply. Using the cropland use data and NPP data for each year, the annual grain production data for each county were retrieved from the statistical yearbooks of each province. The formulae were calculated as follows:

(A5) $$G_{ij}=\frac{NPP_{ij}}{NPP_{j}}\times G_{j}$$

where *G_ij_* is the grain yield of cropland patch i in county *j*, *NPP_ij_* is the *NPP* value of patch *i*, *NPP_j_* is the value of the *NPP* in county *j*, and *G_j_* is the grain yield of county *j*.

### Appendix A.5. Population-Carrying Capacity

The cropland is an important constraint to the population-carrying capacity of a country or region, and the population-carrying function represents the livelihood conditions provided by the agricultural land around the settlement. The cropland landscape is the main carrier of the rural population, and with reference to [34], the calculation of the population carrying function in this paper was corrected using the NDVI of the corresponding year with the following formula:

(A6) $$POP_{x}=\frac{POP_{j}}{NDVI_{j}}\times NDVI_{x}$$

where *POPx* and *POPj* are the rural populations in raster *x* and county *j*, respectively, and *NDVIx* and *NDVIj* are the total vegetation cover indices in raster *x* and county *j*, respectively.

### Appendix A.6. Landscape Aesthetics

Aesthetic landscape services are a combination of nature and society, and the study of the aesthetics of cropland landscapes leads to an appreciation of the aesthetic services that cropland areas perform to enhance human well-being in general. However, this is easily overlooked in the identification of the function of cropland. It is generally accepted that the higher the level, the greater the undulation, and the closer to settlements and roads, the higher the aesthetic value of cropland areas in the landscape [34]. In this paper, we quantified landscape aesthetics by calculating four factors: the surface undulation, degree of aggregation, distance to the nearest road, and distance to the nearest city. The landscape agglomeration was calculated in FRAGSTATS software, while the surface undulation was studied [83] and the road and town distances were quantified by setting distances of 20 km and 50 km, respectively, in ArcGIS software, calculated as follows:

(A7) $$AES=0.3RDLS+0.3CONT+0.25(1-D_{city})+0.15(1-D_{road})\;$$

(A8) $$RDLS=\frac{ALT}{1000}+\frac{H_{dif}\times (1-P_{A}/A)}{500}$$

(A9) $$CONT=100\left(1+\sum_{i=1}^{m}\sum_{j=1}^{m}\frac{p_{ij}\ln p_{ij}}{2\ln m}\right)$$

where *RDLS* is a composite representation of a topographic relief; *H_dif_* is the difference between the highest elevation and the lowest elevation; *ALT* is the average elevation of the region; *D_city_* and *D_road_* are the distances to the nearest towns and roads, respectively; *CONT* is the landscape aggregation index; *PA* and *A* are the flat area and total area of the region, respectively; *m* is the number of patches; *p_ij_* is the proportion of adjacency between image elements *i* and *j*.

### Appendix A.7. Data Standardization

The indicator data values of the function of the cropland in the trough valley area in this study were not at a uniform level of comparison, so the values of each function were dimensionless using the polarization standardization method, and the dimensionless values were between 0 and 1 after polarization.

Positive indicator:

(A10) $$X_{nj}=\frac{x_{nj}-\min(x_{nj})}{\max(x_{nj})-\min(x_{nj})}$$

Negative indicator:

(A11) $$X_{nj}=1-\frac{x_{nj}-\min(x_{nj})}{\max(x_{nj})-\min(x_{nj})}$$

where *X_nj_* is the standardized value of the nth function *j* indicator of cropland in the trough and *x_nj_* is the initial value of the nth function *j* indicator in the grid and for the analysis of the changes in the function of cropland within the trough during the two periods, where the maximum and minimum values represent the maximum and minimum values across the whole study area, respectively.

## Appendix B. Geostatistical Trend Analysis

### Appendix B.1. Geostatistical Trend Analysis

In this paper, based on the ArcGIS platform, CLES values for the karst trough and valley area from 2000 to 2020 were used as the base data, and a multiple polynomial fit was used to generate a 3D perspective network map. Each vertical bar in the figure represents the height and location of CLES measurements within a grid. These points are projected onto east–west and north–south orthogonal planes, which in turn were used to plot a best-fit line and rotate a reasonable perspective angle to reveal in-depth trends in the ecological safety of cropland landscapes in specific directions and to resolve future spatial development patterns of CLES, the specific numerical model of which can be found in [84,85,86].

## Appendix D. Abbreviation List

**Table A1:** Abbreviation list.

Glossary Name	Abbreviation
Annual average temperature	TEM
Annual average precipitation	PRE
Anticlinorium Folds	AF
Cropland landscape ecological security	CLES
Cropland multifunctionality	CM
Cropland coupling coordination degree	CCCD
Distance to country location	DTCL
Distance to main highways	DTMH
Distance to main railways	DTMR
Distance to rivers	DTR
Elevation	DEM
Ejective Folds	EF
Gross national product	GDP
Grain Supply	GS
Hydrothermal vegetation Conditions	HVC
Habitat Quality	HQ
Habitat Connection	HC
Landscape Aesthetics	LA
Natural background	NB
Normalized difference vegetation index	NDVI
Neighborhood enrichment	NE
Night lights	NL
Production Neighborhood environment	PNE
Population Carrying	PC
Slope	SL
Soil Retention	SR
Soil type	ST
Soil organic matter	SOM
Soil erosion intensity	SEI
Socio-economic environment	SE
Tight Folds	TF
Trough-Like Folds	TLF
Topographic relief	TR
